# The Use of Dexamethasone in Bacterial Meningitis in Children and Adults: A Retrospective Analysis

**DOI:** 10.5402/2011/380283

**Published:** 2011-12-28

**Authors:** An-Sophie Cornelis, Said Hachimi-Idrissi

**Affiliations:** Pediatric Critical Care Medicine Department, Universitair Ziekenhuis Brussel, 1090 Brussels, Belgium

## Abstract

Bacterial meningitis is a life-threatening illness that results from bacterial infection of the meninges and is associated with high mortality and morbidity rate, especially when the *Streptococcus pneumoniae* is the causative agent. Dexamethasone as adjunctive therapy to antibiotics does not influence the outcome or the complications in children as well as in adults suffering bacterial meningitis. However, we identified some prognostic parameters in the outcome of bacterial meningitis, and when dexamethasone was given in presence of at least one of 3 poor prognostic CSF parameters (WBC < 1000/mm^3^, glucose < 20 mg/dl, lactate > 10 mg/dl) it substantially improved the outcome.

## 1. Introduction

Bacterial meningitis (BM) is still a worldwide major infectious disease. About 1,2 million cases of BM are estimated to occur annually worldwide with 135.000 deaths [1, 2]. The relative frequency of the various causes of community-acquired meningitis has had a spectacular change over the last 20 years. Since the introduction of *Hib (Haemophilus influenzae type b) *conjugate vaccines in the developed countries, it has resulted in a decline of invasive *Hib* infections by more than 90% [3]. As a consequence *S. pneumoniae* now became the leading species (47%), followed by *N. meningitidis* (25%) and *Listeria monocytogenes* (8%) in community-acquired meningitis in the developed countries [4]. In the beginning of the 19th century, the preantibiotic era, mortality rates for meningitis caused by *S. pneumoniae* and *Hib* were 100% and for *N. meningitidis* 75%–80%. Since the use of third-generation cephalosporins in the eighties, the mortality rates dropped for pneumococcal meningitis to 20–30%, for *Hib* meningitis to 5%, and for meningococcal meningitis to 10%. But since then, there have been little to no change in these rates, in spite of improved diagnostic techniques, the introduction of new antibacterials, adjunctive therapies, and progress in intensive care [4]. With meningococcal meningitis, there is a 10–20% chance of severe sequelae, including permanent hearing loss and mental retardation [5–7]. Pneumococcal meningitis is associated with high mortality and morbidity rates in adults (30%). Whereas neurological complications are the leading cause of death in younger patients, elderly patients die predominantly from systemic complications in pneumococcal meningitis [8]. In children mortality is much less (7.7%–15,3%) but they still have high prevalence of neurological complications (25%) and hearing loss (32%) [9, 10].

It is the host's secondary inflammatory response to the entry of microorganisms into the subarachnoid space, which produces the neuronal damage. Once entered into the subarachnoid space, the bacteria multiply and release proinflammatory and toxic compounds by autolysis or secretion. Antibiotics causes rapid killing of the bacteria in the CSF that effectively sterilizes the meningeal infection but enhances the release of the proinflammatory and toxic compounds that precipitates the cytokine-mediated inflammatory response to the development of cerebral oedema (cytotoxic, interstitial, and vasogenic cerebral oedema [1, 11–13]). Marked increases in intracranial pressure (ICP) can be deleterious in patients with BM by causing cerebral herniation or decreasing cerebral perfusion and can ultimately lead to irreversible brain injury (neuronal damage and apoptosis) and death [1]. As a result, the therapeutic approach to BM has to be widened from eradicating the pathogen with antibiotics to prevention of the detrimental effects of the immune response. Of the various adjunctive therapeutic approaches to prevent this immune response, dexamethasone has been used with success in animal models [14, 15], in clinical trials [16–19], and meta-analyses [20, 21]. Animal studies of BM have shown that dexamethasone is effective in minimizing the inflammatory response secondary to the meningitis infection in the subarachnoid space. Dexamethasone interferes with the production of inflammatory compounds TNF-*α* and IL-1, and limits the increase in CSF lactate and leukocyte concentration. It also reverses development of brain oedema and hence reduces the increase in ICP [14, 15].

We will identify some prognostic parameters that might influence the course of BM as well as the effect of the adjunctive therapy of dexamethasone in these situations on the complications and outcome.

## 2. Patients and Methods

### 2.1. Patients and File Study

From 2000 to 2005, all patients admitted in our hospital with the diagnosis of BM were screened and studied. The patients' data were obtained from the hospital registry. Patients were included according to the following inclusion criteria. The diagnosis of acute bacterial meningitis was based on a positive gram staining or culture of the CSF, the CSF leukocyte count of more than 1000/mm^3^, or the presence of cloudy CSF. If the diagnosis could be made postmortem on autopsy when lumbar puncture was not done, patients were also included. Patients were excluded if they had neonatal meningitis, nosocomial BM, or antecedents of neurosurgery or neurotrauma. We did not exclude patients who received antibiotics in the 48 hours prior to admission, if they met the inclusion criteria.

The following parameters were studied in the patients' files: demographic characteristics, predisposing factors, clinical findings and biological findings, on admission, treatment, neurological, and audiological outcome, mortality, clinical course, and adverse events.

### 2.2. Assessment of Outcome and Prognostic Parameters

The population was studied for the prognostic factors associated with a poor outcome. Poor outcome was defined as mortality, unfavourable outcome eight weeks after discharge (according to the Glasgow outcome scale (GOS)), hearing loss, and focal neurological sequelae (defined as ataxia, hemiparesis, and cranial nerve palsy). The GOS [22] eight weeks after discharge was derived from the medical files. A score of 1 indicates death, 2 a vegetative state (the patient is unable to interact with the environment), 3 severe disability (the patient is unable to live independently but can follow commands), 4 moderate disability (the patient is capable of living independently but unable to return to work or school), and 5 minimal or no disability (the patient is able to return to school or work). A favourable outcome was defined as a score of 5, and an unfavourable outcome as a score of 1 to 4. Hearing loss and focal neurological sequelae information were obtained at discharge and, where available in the medical files, eight weeks after discharge.

### 2.3. Statistical Analysis

For statistical analysis SPSS 12.0 package was used. The unpaired *t*-test was used to analyse the difference in outcome of *S. pneumoniae* meningitis versus *N. meningitis* meningitis in outcome (hypothesized difference = 0). Odds ratio and 95 percent confidence interval were used to quantify the strength of the associations of the prognostic factors for unfavourable versus favourable outcome after 8 weeks and for presence of sequelae at discharge.

Finally ANOVA and Fisher's PLSD test were used to analyse the effect of dexamethasone on the neurological and audiological outcome in high-risk subgroups (CSF parameters of white cell count of less than 1000/mm^3^, glucose of less than 10 mg/dL, and lactate of more than 10 mg/dL; significance level: 5%). A *P* value of less than 0,05 is considered to be significant.

## 3. Results

A number of 138 patients were found with the diagnosis of BM. Twenty patients were withdrawn because of neurosurgery or nosocomial BM and another 6 patients were withdrawn because of neonatal BM. Thirteen patients did not meet the inclusion criteria of a positive gram staining or culture of the CSF, a CSF leukocyte count of more than 1000/mm^3^, or the presence of cloudy CSF. One patient, who had no lumbar puncture, however, with a postmortem diagnosis on autopsy, was included. Consequently we analysed a total number of 99 children and adults who suffered from BM. When the diagnosis was made of BM, based upon clinical signs or laboratory parameters, the patients were treated with appropriate antibiotics. The empirical therapy was ceftriaxone, ampicillin was added to ceftriaxone if the patient was younger than 3 months of age, or older than 60 years of age or in the presence of immunocompromised state. A combination of ceftriaxone plus vancomycin was used if the patient came out of a region with resistant pneumococcal strains or in presence of culture evidence of resistant strains. In the presence of septic shock fluid resuscitation and inotropics were added to the treatment. In case of respiratory failure they were intubated and mechanically ventilated. All patients received gastric protection, by use of H2-antihistaminica.

Nine patients out of the 99 died during the course of their disease. Five of them received dexamethasone while 4 of them did not. The etiological pathogen was identified in 7 of them: six of them had *S. pneumoniae* and 1 had *N. meningitidis*. So the mortality rate for *S. pneumoniae* and for *N. meningitidis* was, respectively, 19% and 2%.

*N. meningitidis* and *S. pneumoniae* were the most frequent causes of acute bacterial meningitis in our study population, respectively, 39% and 32%. There was only one patient in our study population, a girl of 30 months of age, who had *H. influenzae* as causative organism. *Listeria monocytogenes* was not found as causative organism in any patient in our population. Patients with *N. meningitidis* were younger than patients with *S. pneumoniae*, but not significant.

In the total population of children and adults, 49 patients were identified with favourable outcome (GOS of 5), while 17 patients had unfavourable outcome after 8 weeks (GOS of 4 or less). Thirty-three patients were lost for followup. Neurological and or audiological sequelae at discharge were found in 21 patients while 66 patients had no sequelae at discharge (twelve patients had no clinical data in their medical records at discharge).  Table 1 shows the association between unfavourable outcome and Table 2 the association between neurological or audiological sequelae and various clinical and laboratory factors before hospitalisation, on admission, and during hospital stay. There were several factors with no relation to outcome or sequelae, such as age, sex, dexamethasone treatment (see Tables 1 and 2). Various other factors were associated with a negative outcome or with the presence of sequelae. Unfavourable outcome and sequelae were, respectively, 5- and 6-times more frequent if the patients had convulsions at admission, than the patients who had no convulsions. The presence of focal neurological abnormalities at admission was even more associated with worse outcome and sequelae, meaning, respectively, 10- and 11-times more frequent. Neurological and audiological sequelae were also associated with the presence of the triad of symptoms at presentation of neck stiffness, fever, and altered mental status. A positive gram staining or culture for *S. pneumoniae* in the CSF was correlated with worse outcome and sequelae. The laboratory parameters in the CSF of WBC count of less than 1000/mm^3^, glucose less than 20 mg/dL, and lactate more than 10 mmol/L were associated with worse outcome and presence of sequelae. Only the unfavourable outcome was associated with C-reactive protein (CRP) in blood of more than 200 mg/L at admission and hyponatriemia (less than 130 mmol/L) in the first 72 hours after admission. When patients were referred from another hospital they had a higher chance for unfavourable outcome (Table 1).

There was no difference in outcome and presence of sequelae if dexamethasone was used. When dexamethasone was given to patients with WBC of less than 1000/mm^3^ and/or glucose of less than 20 mg/dL and/or lactate of more than 10 mmol/L in the CSF, they had significantly better outcome. Unfavourable outcome after 8 weeks was significantly less in patients that received dexamethasone who had at least one of these 3 conditions. In these conditions there was an 84,7% chance for favourable outcome defined as GOS of 5, and 17,3% chance for unfavourable outcome when dexamethasone was administered. When no dexamethasone was given, there was only a chance of 43,2% of good outcome, and 56,8% chance of unfavourable outcome. The mortality rate was not significantly affected by use of dexamethasone in one of these conditions (*P* = 0,12).

## 4. Discussion

In accordance with the literature the common causative organisms of BM in our study were *S. pneumoniae* and *N. meningitidis*. The mortality rate for *S. pneumoniae* and for *N. meningitidis* was slightly lower than reported in the literature [4, 20]. The overall outcome was worse in BM with *S. pneumoniae* than with *N. meningitidis* and this is comparable to the literature data [5, 8–10].

As shown in previous studies the presence of convulsions, focal neurological abnormalities, or impaired consciousness at admission are strongly associated with adverse outcome and sequelae [8, 23–28]. While the triad of symptoms (neck stiffness, altered mental status, and fever) was only present in 25 percent of the patients, this triad was associated with neurological or audiological sequelae, as comparable to the literature [23]. However, our population consisted of both children and adults, and the low rate of presence of the classic triad at presentation was often observed in the children group. In the age group younger than 12 months neckstiffness is not always an interpretable clinical sign. When the patients had petechial rash on admission, there was a positive association with good outcome and the absence of sequelae, as in the literature data [23, 29]. Reflecting that petechiae are more associated with *N. meningitidis*, and this pathogen has a better outcome in bacterial meningitis, compared to *S. pneumoniae*. A low CSF WBC count was associated with an adverse outcome. This was due to a fulminant meningococcal sepsis and meningitis. This association has been described earlier [8, 23, 26, 27, 30]. The laboratory parameters in the CSF, glucose less than 20 mg/dL, and lactate more than 10 mmol/L were associated with worse outcome and presence of sequelae, this was also found in adults and children literature [23, 29, 31, 32]. In our study unfavourable outcome was also associated with C-reactive protein (CRP) in blood of more than 200 mg/L on admission and hyponatriemia (less than 130 mmol/L) in the first 72 hours after admission.

We found that dexamethasone was more effective in situations were WBC count was low in the CSF (<1000/mm^3^). Although a high WBC count in the CSF is correlated with a high inflammatory response, and dexamethasone interferes with this inflammatory reaction. A low WBC count in the CSF probably means excessive bacterial growth and insufficient leukocyte response, hence increased complications [8, 23, 26, 27, 30], explaining our results. In accordance with our study, CSF levels of WBC count less than 1000/mm^3^, glucose less than 20 mg/dL, and lactate more than 10 mmol/L in CSF dexamethasone treatment, were able to reduce complications. But further studies are needed to support these findings. A recent published trial of BM in adults [19] also showed that patients with a GCS between 8 to 11 on admission have a better neurological outcome when dexamethasone was administered.

The above prognostic parameters, together with the evidence of beneficial effect of dexamethasone in pneumococcal meningitis, might help to identify those patients who will utmost beneficiate from dexamethasone therapy.

Routine use of dexamethasone is now increasingly used in children and adults with BM. Although benefit was not proven in all etiological subgroups (mainly *N. meningitidis*), failure to demonstrate an effect is more likely due to the limited power from low event rates and limited size of the studied populations in the literature. The pathophysiological mechanisms that result in CNS damage seems to be similar in the different etiological organisms. They all stimulate the production of TNF-*α* and IL-1, which are suppressed by administration of dexamethasone. Therefore why should dexamethasone not have the same beneficial effect in meningococcal meningitis as in *Hib *and *S. pneumoniae* meningitis?

*S. pneumoniae* as an etiological cause and a GCS on admission of 8 to 11 have already been identified as situations where dexamethasone is more beneficial. [19].

In this retrospective study we found that in presence of at least one of 3 CSF parameters (WBC < 1000/mm^3^, glucose < 20 mg/dL, and lactate > 10 mg/dL) dexamethasone would have the most beneficial effect. However, in the clinical setting, dexamethasone and antibiotics are most frequently given when bacterial meningitis is suspected before the causative germ is known.

In view of this retrospective analysis with its limitations, more studies are needed to confirm the results and, moreover, to detect a possible beneficial effect of dexamethasone therapy in patients with meningococcal meningitis. The prospect of clinical trials in children, already limited by small case numbers, will be further reduced since the decline in invasive pneumococcal and meningococcal (serogroup C) infections in children as a result of the implementation of the vaccines.

## 5. Conclusions

We identified 3 prognostic parameters (WBC < 1000/mm^3^, glucose < 20 mg/dL, and lactate > 10 mg/dL) which seems to be associated with poor outcome in BM. Administration of adjunctive dexamethasone therapy in these condition would have most beneficial effect on the outcome.

## Figures and Tables

**Table 1:** Prognostic factors for unfavourable versus favourable outcome after 8 weeks.

	Unfavourable (*n* = 17)	Favourable (*n* = 49)	Odds ratio (95% CI)*
Age (yr)—mean ± SD	21 ± 30	20 ± 32	—
Males—%	65	51	—
Duration symptoms before admission (hr)—mean ± SD	45 ± 45	34 ± 41	—
Duration symptoms before admission <24 hrs—no./tot. no. (%)	10/17 (59)	36/48	0.48 (0.15–1.53)
Received dexamethasone—no./tot. no. (%)	11/17 (65)	33/49 (67)	0.89 (0.18–2.84)
Received AB in last 48 hr—no./tot. no. (%)	4/17 (24)	9/48 (19)	1.33 (0.35–5.06)
Coexisting conditions—no./tot. no. (%)			
Otitis/sinusitis	2/17 (12)	15/49 (31)	0.30 (0.061–1.49)
Pneumoniae	3/17 (18)	4/49 (8)	2.41 (0.48–12.09)
Immunocompromise	4/17 (24)	3/49 (6)	4,72 (0,96–23,8)
Convulsions on admission—no./tot. no. (%)	7/17 (41)	6/49 (12)	**5,0 (1,4**–**18,2)**
Findings on admission—no./tot. no. (%)			
Normal consciousness	2/17 (12)	31/49 (63)	**0,07 (0,02**–**0,38)**
GCS score < 8	4/10 (40)	2/40 (5)	**12.67 (1.89–84.97)**
Focal neurological signs	5/17 (29)	2/49 (4)	**9,8 (1,69**–**56,8)**
Temperature > 38°C	13/17 (76)	34/48 (71)	1.34 (0.37–44.82)
Triad (neck stiffness, fever, and altered mental status)	4/11 (36)	10/35 (28)	1.43 (0.34–5.97)
Petechiae	1/17 (6)	21/49 (43)	**0,08 (0,01**–**0,68)**
CSF culture—no./tot. no. (%)			
* S. pneumonia*	12/17 (70)	15/49 (31)	**5,44 (1,62**–**18,2)**
* N. meningitidis*	3/17 (18)	17/49 (35)	0,4 (0,102–1,60)
Other bacteria	1/17 (6)	8/49 (16)	0.32 (0.037–2.77)
Negative culture	1/17 (6)	9/49 (18)	0.28 (0.032–2.37)
CSF parameters			
Positive bacterial culture—no./tot. no. (%)	16/16 (100)	38/49 (78)	—
WBC (/mm^3^)—mean ± SD	3401 ± 7211	6057 ± 8156	—
WBC < 1000/mm^3^—no./tot. no. (%)	10/13 (77)	15/49 (31)	**7,6 (1,82**–**31,4)**
Proteins (mg/dL)—mean ± SD	558 ± 557	305 ± 263	—
Proteins > 500 mg/dL—no/tot no (%)	6/13 (46)	12/49 (24)	2,64 (0,74–9,39)
Glucose (mg/dL)—mean ± SD	18,5 ± 21,8	35,6 ± 25,1	—
Glucose < 20 mg/dL—no/tot no (%)	11/13 (85)	20/49 (41)	**7,98 (1,59**–**39,9)**
Glucose CSF/serum ratio (%)—mean ± SD	16 ± 14	28 ± 20	—
Lactate (mmol/L)—mean ± SD	12,3 ± 4,9	8,0 ± 5,0	—
Lactate > 10 mmol/L—no./tot. no. (%)	7/9 (78)	15/44 (34)	**6,77 (1,25**–**36,7)**
Positive blood culture, no./tot. no. (%)	9/16 (56)	22/48 (56)	1.52 (0.49–4.75)
Blood parameters			
Hb** (g/dL)—mean ± SD	10,8 ± 2,4	12,0 ± 1,9	—
Platelets (×10^3^/mm^3^)—mean ± SD	269 ± 143	280 ± 148	—
CRP*** (mg/L)—mean ± SD	258 ± 119	176 ± 128	—
CRP > 200 mg/L—no./tot. no. (%)	12/17 (70)	19/49 (39)	**3,78 (1,15**–**12,5)**
Hyponatremia** in first 72u—no./tot. no. (%)	4/17 (24)	2/49 (4)	**7,23 (1,19**–**43,9)**
Referred from an other hospital—no./tot. no. (%)	10/17 (59)	9/49 (18)	**6,35 (1,9**–**21,2)**

*CI = confidence interval, **Hb = haemoglobin, ***CRP = C-reactive protein.

**Table 2:** Prognostic factors for neurological or audiological sequelae at discharge.

	Neuro/audio sequel (*n* = 21)	No sequel (*n* = 66)	Odds ratio (95% CI)
Age (yr)—mean ± SD	19 ± 26	15 ± 20	—
Males—%	57	47	—
Duration symptoms before admission (hr)—mean ± SD	44 ± 51	33 ± 39	—
Duration symptoms before admission < 24 hrs—no./tot. no. (%)	14/21 (67)	49/64 (76)	0.61 (0.21–1.80)
Received Dexamethasone—no./tot. no. (%)	16/21 (76)	46/66 (70)	1.39 (0.45–4.32)
Received AB in last 48 hr—no./tot. no. (%)	5/21 (24)	7/65 (11)	2.59 (0.72–9.26)
Coexisting conditions—no/tot no (%)			
Otitis/sinusitis	6/21 (28)	11/66 (17)	2.00 (0.66–6.30)
Pneumoniae	3/21 (14)	3/66 (4)	3.5 (0.65–18.85)
Immunocompromise	0/21	5/66 (8)	—
Convulsions on admission—no./tot. no. (%)	7/21 (33)	5/66 (8)	**6.10 (1.69–22.08)**
Findings on admission—no./tot. no. (%)			
Normal consciousness	7/21 (33)	45/64 (70)	**0.21 (0.074–0.60)**
GCS score < 8	0/11	3/50 (6)	**—**
Focal neurological signs	3/21 (14)	1/66 (2)	**10.83 (1.06–110.53)**
Temperature > 38°C	17/21 (81)	44/65 (68)	2.03 (0.61–6.78)
Triad (neck stiffness, fever, and altered mental status)	7/13 (54)	9/52 (17)	**5.57 (1.51–20.57)**
Petechiae	4/21 (19)	36/66 (54)	**0.20 (0.060–0.64)**
CSF culture—no./tot. no. (%)			
* S. pneumoniae*	14 (67)	11 (17)	**10 (3.28–30.48)**
* N. meningitidis*	3 (14)	33 (50)	**0.17 (0.045–0.62)**
Other bacteria	2 (10)	7 (11)	0.84 (0.17–4.64)
Negative culture	2 (10)	15 (23)	0.36 (0.075–1.72)
CSF parameters			
Positive bacterial culture—no./tot. no. (%)	18/21 (86)	50/66 (76)	1.92 (0.50–7.37)
WBC (/mm^3^)—mean ± SD	2805 ± 4891	5763 ± 7845	—
WBC < 1000/mm^3^—no./tot. no. (%)	11/18 (61)	21/64 (33)	**3.22 (1.09–9.49)**
Proteins (mg/dL)—mean ± SD	325 ± 248	262 ± 238	—
Proteins > 500 mg/dL—no./tot. no. (%)	5/18 (28)	12/61 (20)	1.57 (0.47–5.26)
Glucose (mg/dL)—mean ± SD	22,2 ± 18,3	42,0 ± 26,3	—
Glucose < 20 mg/dL—no./tot. no. (%)	11/18 (61)	20/62 (32)	**3.30 (1.11–9.78)**
Glucose CSF/serum ratio (%)—mean ± SD	21 ± 17	33 ± 22	**—**
Lactate (mmol/L)—mean ± SD	9,9 ± 4,4	7,9 ± 6,3	**—**
Lactate > 10 mmol/L—no./tot. no. (%)	10/16 (62)	12/48 (25)	**5 (1.50–16.68)**
Positive blood culture—no./tot. no. (%)	9/20 (45)	33/63 (52)	0.74 (0.27–2.04)
Blood parameters			
Hb (g/dL)—mean ± SD	11 ± 2	12 ± 2	—
Platelets (×10^3^/mm^3^)—mean ± SD	291 ± 141	270 ± 136	—
CRP (mg/L)—mean ± SD	218 ± 134	172 ± 128	—
CRP > 200 mg/L—no./tot. no. (%)	11/21 (52)	23/66 (35)	2.06 (0.76–5.56)
Hyponatremia in first 72u—no./tot. no. (%)	3/21 (14)	3/66 (4)	3.5 (0.65–18.85)
Referred from another hospital—no./tot. no. (%)	7/21 (33)	27/66 (41)	0.72 (0.26–2.03)
